# Targeted therapies in children with renal cell carcinoma (RCC): An International Society of Pediatric Oncology—Renal Tumor Study Group (SIOP‐RTSG)‐related retrospective descriptive study

**DOI:** 10.1002/cam4.6782

**Published:** 2023-12-22

**Authors:** Julia Sprokkerieft, Justine N. van der Beek, Filippo Spreafico, Barbara Selle, Estelle Thebaud, Tanzina Chowdhury, Jesper Brok, Gábor Ottóffy, Xiaofei Sun, Gema L. Ramírez Villar, Garik Sagoyan, Heidi Segers, Dimitrios Doganis, Annalisa Serra, Lauriane Lemelle, Norbert Graf, Arnauld C. Verschuur, Godelieve A. M. Tytgat, Marry M. van den Heuvel‐Eibrink

**Affiliations:** ^1^ Department of Pediatric Oncology Princess Máxima Center for Pediatric Oncology Utrecht The Netherlands; ^2^ Pediatric Oncology Unit, Department of Medical Oncology and Hematology Fondazione IRCCS Istituto Nazionale dei Tumori Milano Italy; ^3^ Hopp Children's Cancer Center Heidelberg (KiTZ) & Division of Pediatric Neurooncology German Cancer Research Center (DKFZ) and German Cancer Consortium (DKTK) Heidelberg Germany; ^4^ Hôtel Dieu, CHU de Nantes Nantes France; ^5^ Pediatric Oncology, Great Ormond Street Hospital for Children NHS Foundation Trust London UK; ^6^ Department of Pediatric Hematology and Oncology, Rigshospitalet Copenhagen University Hospital Copenhagen Denmark; ^7^ Department of Pediatrics, Medical School University of Pécs Pécs Hungary; ^8^ Department of Pediatric Oncology Sun Yat‐sen University Cancer Center Guanghzou China; ^9^ Department of Pediatric Oncology Hospital Universitario Virgen del Rocío Seville Spain; ^10^ N.N. Blokhin National Medical Research Center of Oncology Moscow Russia; ^11^ Department of Pediatric Hemato‐Oncology University Hospitals Leuven and Catholic University Leuven Leuven Belgium; ^12^ Oncology Department P&A Kyriakou Children's Hospital Athens Greece; ^13^ Dipartimento di Onco‐Ematologia e Medicina Trasfusionale IRCCS Ospedale Bambino Gesù Roma Italy; ^14^ SIREDO Oncology Center (Care, Innovation and Research for Children and AYA with Cancer) Institut Curie Paris France; ^15^ Department of Pediatric Oncology and Hematology Saarland University Homburg Germany; ^16^ Department of Pediatric Hematology‐Oncology, Hôpital d'Enfants de la Timone, APHM Marseille France

**Keywords:** MiT family translocation, pediatric, renal cell carcinoma, targeted therapy, treatment response

## Abstract

**Introduction:**

Introduction: Renal cell carcinoma (RCC) is a very rare pediatric renal tumor. Robust evidence to guide treatment is lacking and knowledge on targeted therapies and immunotherapy is mainly based on adult studies. Currently, the International Society of Pediatric Oncology–Renal Tumor Study Group (SIOP‐RTSG) 2016 UMBRELLA protocol recommends sunitinib for metastatic or unresectable RCC.

**Methods:**

This retrospective study describes the effects of tyrosine kinase inhibitors (TKI), anti‐programmed cell death 1 (PD‐(L)1) monoclonal antibodies, and immunotherapeutic regimens in advanced‐stage and relapsed pediatric RCC.

**Results:**

Of the 31 identified patients (0–18 years) with histologically proven RCC, 3/31 presented with TNM stage I/II, 8/31 with TNM stage III, and 20/31 with TNM stage IV at diagnosis. The majority were diagnosed with translocation type RCC (MiT‐RCC) (21/31) and the remaining patients mainly presented with papillary or clear‐cell RCC. Treatment in a neoadjuvant or adjuvant setting, or upon relapse or progression, included mono‐ or combination therapy with a large variety of drugs, illustrating center specific choices in most patients. Sunitinib was often administered as first choice and predominantly resulted in stable disease (53%). Other frequently used drugs included axitinib, cabozantinib, sorafenib, and nivolumab; however, no treatment seemed more promising than sunitinib. Overall, 15/31 patients died of disease, 12/31 are alive with active disease, and only four patients had a complete response. The sample size and heterogeneity of this cohort only allowed descriptive statistical analysis.

**Conclusion:**

This study provides an overview of a unique series of clinical and treatment characteristics of pediatric patients with RCC treated with targeted therapies.

## Graphical abstract

This is the first SIOP‐RTSG retrospective study that reports response‐ and survival data of pediatric patients with renal cell carcinoma (RCC) who were treated with targeted therapy. Thirty‐one patients with advanced‐stage RCC were treated with 17 different treatment regimens, highlighting current treatment heterogeneity. This descriptive study emphasizes the need for continued registration of pediatric RCC and uniform treatment with sunitinib until other targeted therapies have been further investigated, for instance using RCC organoids.

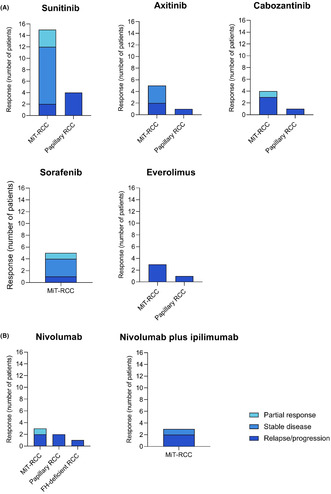

**Abbreviations**
AIEOP: Associazione Italiana Ematologia Oncologia Pediatrica
CRF: Case report form
IMPORT: Improving Populations Outcome of Renal Tumors of Childhood
MiT: Microphthalmia transcription factor
MiT‐RCC: MiT family translocation renal cell carcinoma
PD‐(L)1: Programmed death‐ligand 1
RCC: Renal cell carcinoma
RECIST: Response Evaluation Criteria in Solid Tumors
RTSG: Renal Tumor Study Group
SIOP: International Society of Pediatric Oncology
TFE3: Transcription factor E3
TKI: Tyrosine kinase inhibitor
TNM: Tumor‐node‐metastasis
TTP: Time‐to‐progression
VEGFR: Vascular endothelial growth factor receptor
WHO: World Health Organization

## 1 INTRODUCTION

Renal cell carcinoma (RCC) is a type of kidney cancer that is rarely observed in children.^1^ RCC consists of several histological subtypes, with clear cell and papillary subtypes being the most common in adults, whereas in children these subtypes comprise less than half of patients.^2^, ^3^ Approximately half of pediatric patients are diagnosed with translocation type RCC (MiT‐RCC) that involve the microphthalmia transcription factor (MiT).^4^ The most frequently found translocations affect transcription factor E3 (TFE3) and transcription factor EB (TFEB), with genes localized on chromosome Xp11.2 and 6p21, respectively.^3^ MiT‐RCC was first introduced in the World Health Organization (WHO) 2004 Classification of Tumors of the Urinary System and Male Genital Organs. The Vancouver classification, last updated in 2013, also included MiT‐RCC as a subtype, and has contributed to the inclusion of this subtype in the WHO 2016 classification.^5^, ^6^ It has been suggested that the outcome of this subtype may be worse.^7^, ^8^ Currently, sunitinib is recommended by the International Society of Pediatric Oncology—Renal Tumor Study Group (SIOP‐RTSG) for nonlocalized MiT‐RCC.^9^ However, while it is anticipated that treatment of pediatric patients with worse prognosis may follow rapid development of novel agents in adults, it may well be the case that pediatric RCC, especially MiT‐RCC, requires different targeted treatment approaches given the lack of genomic overlap.^8^, ^10^, ^11^

In adults, multiple systemic therapies comprising immunotherapy and tyrosine kinase inhibitors (TKI) have been widely implemented for patients with metastatic disease.^12^ Novel agents, including axitinib, are emerging next to sunitinib, cabozantinib, and pazopanib, which are current first‐line treatment options for poor risk clear‐cell RCC.^1^, ^12^, ^13^, ^14^, ^15^, ^16^, ^17^, ^18^ Combination treatments can consist of combinations of vascular endothelial growth factor receptor‐TKI (VEGFR‐TKI), anti‐programmed cell death 1 (anti‐PD1) monoclonal antibodies, and other immunotherapeutic drugs.^19^, ^20^ Ultimately, decision‐making is dependent on multiple factors, including disease stage, validated prognostic factors, patient age, and previous lines of treatment.^21^ By contrast, the use and effect of targeted therapy on pediatric RCC, and especially MiT‐RCC, has not been extensively investigated due to very few patients.^8^, ^22^ However, axitinib and nivolumab are considered promising drugs, and this combination is currently being studied in the Children Oncology Group (COG) AREN1721 phase II trial for patients aged 12 months and older with an unresectable or metastasized MiT‐RCC.^14^, ^23^

Survival rates for metastatic‐ and relapsed pediatric patients with RCC are poor. Following SIOP‐RTSG (93‐01, 2001, Improving Population Outcomes for Renal Tumours of Childhood (UK‐IMPORT)) protocols, children with metastatic disease have a 5‐year survival of 45.6% with the current treatment regimens.^10^ This illustrates the need for international studies and development of new treatment options.^24^ It is crucial that the effect of these agents in pediatric RCC is determined, since children may have a different response and tolerance to drugs than adults. Furthermore, the efficacy of novel agents specific for MiT‐RCC is not extensively studied in adults, since not MiT‐RCC but clear‐cell RCC is the main histology found in these patients and therefore, most frequently included in studies.^2^, ^25^ This is the first international retrospective series from the SIOP‐RTSG that describes the effect of novel targeted drugs. Main focusses were to report the clinical presentation, objective response, and clinical benefit of pediatric RCC patients treated with TKIs, PD‐(L)1 drugs, and other novel targeted regimens.

## 2 PATIENTS AND METHODS

### 2.1 Patient collection

Here we describe a retrospective cohort study of pediatric patients aged 0–18 years with histologically confirmed RCC and included in SIOP‐RTSG, AIEOP (Associazione Italiana Ematologia Oncologia Pediatrica), and UK‐IMPORT protocols from 1993. All patients treated with TKIs, PD‐(L)1‐inhibitors, or other novel targeted agents in a neo‐adjuvant, adjuvant, relapse, and/or progression setting, and registered in SIOP 93‐01, SIOP 2001, 2016 UMBRELLA, UK‐IMPORT and AIEOP studies, were included. Patients were not excluded in case they were treated with chemotherapy at some point during treatment in addition to targeted therapy. Informed consent was obtained from parents of included patients prior to treatment, according to national law and regulations.

### 2.2 Data collection

In addition to information in the respective databases, an ad hoc case report form (CRF) was constructed to retrospectively collect study specific data (Table S1). The CRF was designed to collect treatment and response data from diagnosis until the latest treatment regimen. This CRF was sent to coordinating investigators in the countries participating in the SIOP and AIEOP studies.

### 2.3 Histopathology

Patients were classified according to the WHO 2004 Classification of Tumors of the Urinary System and Male Genital Organs when possible.^7^, ^26^ In addition, some patients were classified according to the WHO 2016 Classification of Tumors of the Urinary System and Male Genital Organs.^6^ The remaining patients were classified following previous histological classifications. Central review was not conducted for all patients.

### 2.4 Staging

Cancer staging was primarily pursued according to the American Joint Committee on Cancer TNM staging system.^27^, ^28^ Although the CRF asked for detailed TNM cancer staging, due to the absence of specific TNM data, only the overall TNM stage was reported in this descriptive study.

### 2.5 Response assessment

In most patients, the objective response (OR) was classified as complete response (CR), partial response (PR), stable disease (SD), or progressive disease (PD). If available, radiological guidelines were based on size, and the tumor response was determined through Response Evaluation Criteria in Solid Tumors (RECIST) criteria, WHO criteria, or other guidelines. Furthermore, patients achieving complete remission after surgery were described as surgical complete response (SCR). All treatment approaches with available objective response data were reported in the results. When response data could not be traced back, the response was described as “not specified”. One “treatment approach” defined the start of treatment with a given drug or drug combination until a response was measured that led to a new treatment approach based on that response measurement. Sometimes, sequential response assessments were available for a given treatment approach, with a response assessment during treatment and at disease‐progression. For unambiguous and clear reporting, the best response was reported. Although this study focused on targeted therapy, response data were available from several patients who received cytostatic drugs next to targeted therapy. The best way to treat RCC is still uncertain and in need of improvement and therefore, all available data were included in the analysis.

Outcome measurements other than objective response were difficult to interpret due to incomplete reporting. The heterogeneity and, thus, small sample size of the administered treatment regimens makes that for many drugs, time‐to‐progression (TTP) does not completely and sufficiently envision the treatment response. However, TTP is a very useful metric to evaluate drug performance and will therefore be available in Figure S1.

### 2.6 Statistical analysis

A descriptive analysis was performed, as the small number of patients, heterogeneous treatment‐ and outcome data, and relatively few events did not allow for a proper statistical analysis.

## 3 RESULTS

### 3.1 Patient characteristics

In total, 31 pediatric patients, originating from 11 countries, that had been treated with targeted therapies, were retrospectively identified between October 2010 and October 2021 (Table 1). Twelve out of 31 patients (38.7%) were male. The median age at diagnosis was 11.4 years (range 12–206 months). One patient with renal medullary carcinoma (RMC) suffered from known sickle cell trait, while no other underlying genetic predisposition syndromes have been diagnosed in the remaining patients.

**TABLE 1:** Description of patient‐ and tumor characteristics.

No.	Age at diagnosis (months)	Sex	Stage at diagnosis	TFE‐assessment	Histology at diagnosis	Surgery	Complete Resection	No. positive LN/LN sampled	No. of relapses/progressions	Outcome
1	197	M	II	No	MiT‐RCC	Total nephrectomy	*NS*	2/2 (R)	2	ED
2	178	F	II	Yes	MiT‐RCC	Total nephrectomy	No	4/4 9/10 (R) 15/18 (R)	2	ED
3	84	M	III	Yes	MiT‐RCC	Total nephrectomy	Yes	3/3 2/63	5	DOD
4	151	F	III	Yes	MiT‐RCC	Total nephrectomy	No	5/13	1	ED
5	123	M	III	Yes	MiT‐RCC	Total nephrectomy	Yes	12/22	2	DOD
6	77	F	III	Yes	MiT‐RCC	Total nephrectomy	Yes	6/9 15/26	2	ED
7	94	M	III	Yes	MiT‐RCC	Total nephrectomy	Yes	1/3	3	DOD
8	143	M	III	Yes	MiT‐RCC	Total nephrectomy	*NS*	1/1	0	NED
9	180	M	III	Yes	MiT‐RCC	Pre‐op setting	–	–	0	ED
10	180	F	IV	Yes	MiT‐RCC	Total nephrectomy	Yes	2/5	3	DOD
11	12	F	IV	Yes	MiT‐RCC	Total nephrectomy	No	*NS*	8	DOD
12	84	F	IV	Yes	MiT‐RCC	Total nephrectomy	No	17/22	6	DOD
13	206	M	IV	Yes	MiT‐RCC	Total nephrectomy	No	4/9	1	ED
14	178	F	IV	Yes	MiT‐RCC	Total nephrectomy	Yes	6/6	2	DOD
15	204	F	IV	Yes	MiT‐RCC	Total nephrectomy	Yes	1/38	2	DOD
16	48	F	IV	Yes	MiT‐RCC	Total nephrectomy	Yes	6/20	0	ED
17	180	M	IV	Yes	MiT‐RCC	Total nephrectomy	No	3/3	1	ED
18	192	F	IV	Yes	MiT‐RCC	Total nephrectomy (R)	Yes (R)	1/1	1	NED
19	144	F	IV	Yes	MiT‐RCC	Bilateral nephrectomy	Yes	*NS*	2	ED
20	135	F	IV	Yes	MiT‐RCC	No surgery	–	1/1	1	DOD
21	26	F	IV	Yes	MiT‐RCC	No surgery	–	–	7	DOD
22	196	M	IV	Yes	Papillary	Total nephrectomy	Yes	3/*NS*	2	DOD
23	144	F	IV	Yes	Papillary	Total nephrectomy	Yes	Yes/*NS*	1	DOD
24	126	M	IV	Yes	Papillary	Total nephrectomy	No	3/14	–	ED
25	156	F	IV	Yes	Papillary	Total nephrectomy	No	–	1	DOD
26	156	F	IV	No	Papillary	No surgery	–	–/*NS*	0	ED
27	153	F	IV	No	RMC	Total nephrectomy	Yes	1/1	–	DOD
28	174	M	IV	No	RMC	No surgery	–	–/*NS*	1	DOD
29	185	F	I	Yes	Clear cell	Total nephrectomy	Yes	3/3 (R)	1	NED
30	84	M	III	NS	Clear cell	Total nephrectomy	Yes	1/7	0	NED
31	NS	F	IV	Yes	FH deficient	Pre‐op setting	–	–	0	ED

Abbreviations: (R), relapse; DOD, dead of disease; ED, evidence of disease; F, female; FH, fumarate hydratase‐deficient; LN, lymph nodes; M, male; MiT‐RCC, translocation RCC; NED, no evidence of disease; No., number; NS, not specified; RMC, renal medullary carcinoma.

### 3.2 Tumor characteristics

The majority of the patients were diagnosed with advanced‐stage disease: 8/31 had stage III and 20/31 had stage IV disease upon diagnosis. One patient had stage I disease and two patients had stage II disease upon diagnosis, and all were treated with targeted therapy in an adjuvant setting or upon relapse or progression (Table 1). One patient with MiT‐RCC presented with bilateral disease. Regional lymph node status was available for 23 patients and in all these patients, lymph nodes were found to be infiltrated with tumor cells. The few reported sites of metastasis included lymph nodes, bones, lung, liver, brain, or other intra‐abdominal localizations.

### 3.3 Histology

Twenty‐six out of 31 patients were tested for MiT‐RCC, of which 21/26 had a TFE‐translocation (Table 1). TFE‐assessment techniques were unspecified for five patients, whereas 21 patients were assessed with either fluorescence in situ hybridization (FISH) (9/21), immunohistochemistry (IHC) (7/21), cytogenetics (1/21), both FISH and IHC (2/21), IHC and RT‐PCR (1/21), or IHC and karyotype testing (1/21). As for the remaining patients, 5/31 had papillary RCC, 2/31 had renal medullary carcinoma (RMC), 2/31 had clear‐cell RCC, and 1/31 had fumarate hydratase (FH) deficient RCC.

### 3.4 Surgical approach

In total, 25/31 patients (stage I, *n* = 1; stage II, *n* = 2; stage III, *n* = 7; stage IV, *n* = 15) underwent total nephrectomy, of which one patient underwent bilateral nephrectomy (Table 1). One patient with stage IV disease underwent a complete nephrectomy after progression of both the primary tumor and metastases. Complete resection without positive margins was achieved in 15/25 patients. Meanwhile, 2/31 patients were still under follow‐up and had surgery planned at the latest timepoint of data inclusion. In 4/31 patients, surgery was never performed.

### 3.5 Neoadjuvant treatment and response

**FIGURE 1:**
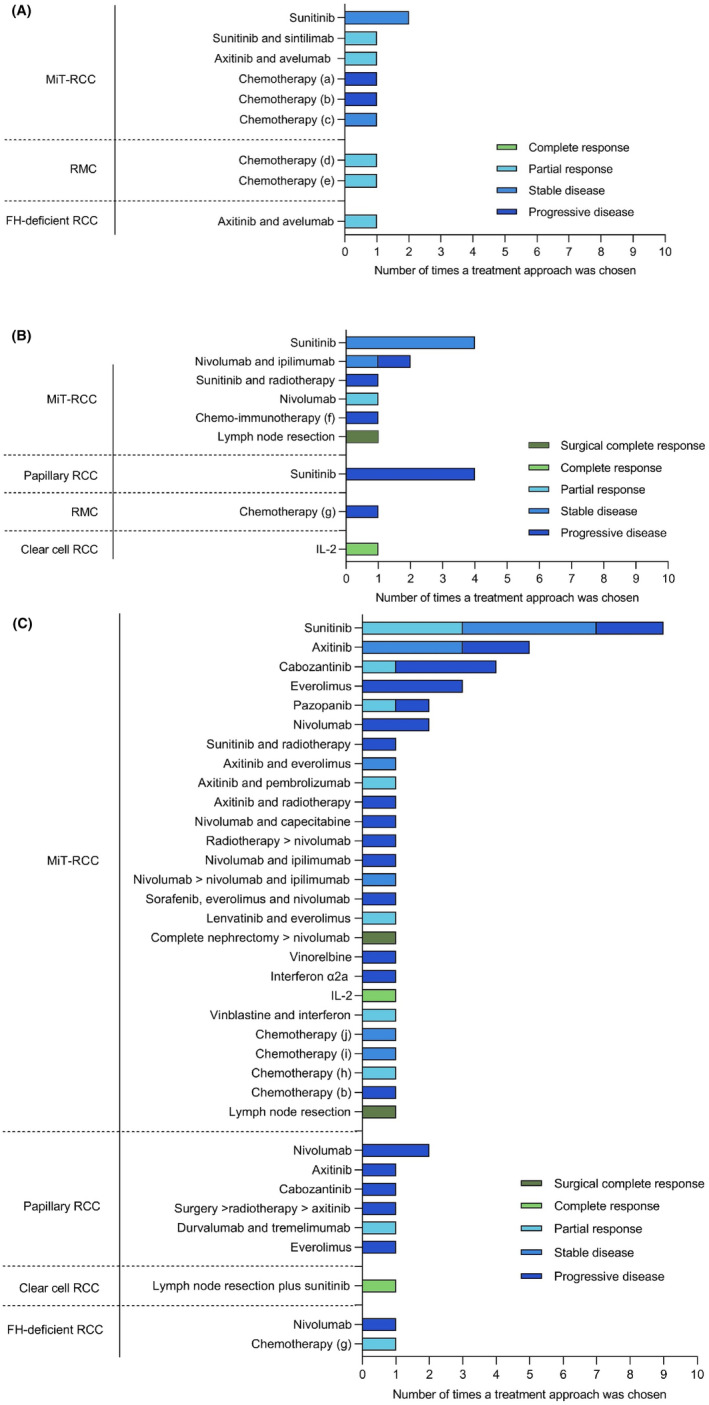
One “treatment approach” defines the start of treatment with a certain drug or drug combination until a response measurement leading to a new treatment approach based on the response measurement (i.e., treatment with a different drug). The best response was reported. (a): capecitabine, isotretinoin plus IL‐2, IFN‐a2a and DGCiN98; (b): actinomycin‐D, vincristine and doxorubicin; (c): gemcitabine and oxaliplatin; (d): cisplatin, paclitaxel and doxorubicin; cisplatin, gemcitabine, ifosfamide; cisplatin, paclitaxel, gemcitabine or bortezomib, carboplatin and gemcitabine; (e): cisplatin, paclitaxel and gemcitabine. (f): IFN‐α, IL‐2, capecitabine and isotretinoin; (g): cisplatin, paclitaxel and gemcitabine/bortezomib, carboplatin and gemcitabine; (h): ifosfamide, cisplatin and doxorubicin; (I): topotecan plus carboplatin; (j): cisplatin and 5‐fluorouracil; (k): olaparib plus irinotecan.

In this cohort, 13/31 patients received neoadjuvant treatment. In 10/13 patients, a response measurement was carried out. Of all patients receiving neoadjuvant treatment, 7/13 had stage IV disease before undergoing nephrectomy, whereas 2/13 (stage III, *n* = 1; stage IV, *n* = 1) patients still have to undergo nephrectomy and 4/13 (stage IV, *n* = 4) patients did not undergo nephrectomy because it was not possible (Figure 1, Table 2, Table S1). In total, 5/13 patients received subsequent adjuvant treatment, 10/13 patients eventually had a relapse or progressed, and 7/13 patients died of disease. The TTP of these 13 patients is shown, if available, in Figure S1A.

**TABLE 2:** Overview of different types of immunotherapies and targeted therapies in this case series.

TKI monotherapy	Immuno‐monotherapy	TKI combination therapy	TKI combined with immunotherapy	Immuno‐combination therapy
Sunitinib	Nivolumab	Axitinib and everolimus	Sunitinib and sintilimab	Nivolumab and ipilimumab
Axitinib	IL‐2	Sorafenib and everolimus	Axitinib and pembrolizumab	Durvalumab and tremelimumab
Cabozantinib		and nivolumab	Axitinib and avelumab	
Pazopanib		Lenvatinib and everolimus		
Sorafenib				
Everolimus				
Bortezomib				

The two patients with RMC underwent neoadjuvant treatment and both showed a partial response to chemotherapy (Figure 1A, Table S1). These chemotherapeutic regimens consisted of cisplatin, paclitaxel, doxorubicin, gemcitabine, ifosfamide, bortezomib, and carboplatin in one patient (TTP: 3.7 months and 6.3 months), and cisplatin, paclitaxel, and gemcitabine in the other patient (TTP: 7.5 months). Three patients with MiT‐RCC had progressive disease (*n* = 2) or stable disease (*n* = 1) after treatment with different chemotherapeutic regimens. One of these patients was treated following SIOP 2001 with chemotherapy (actinomycin‐D, vincristine, and doxorubicin), which resulted in progressive disease (TTP: 0.7 months). The two other patients were treated with the combinations of irinotecan plus olaparib or gemcitabine plus oxaliplatin and had progressive‐ or stable disease, respectively (Figure 1A, Table S1).

Treatment also consisted of TKI monotherapies or combinations of TKIs and immunotherapies. Sunitinib was administered to three patients with MiT‐RCC and resulted in stable disease in two patients (TTP: 6.5–11 months), whereas the response for one patient was unavailable. Two other patients with MiT‐RCC had a partial response after the combination of sunitinib plus sintilimab (TTP: 3.6 months) or axitinib plus avelumab. Similarly, the FH‐deficient RCC had a partial response to axitinib plus avelumab. Lastly, the papillary RCC progressed under sunitinib (TTP: 2.8 months) (Figure 1A, Table S1).

### 3.6 Adjuvant treatment and response

After nephrectomy, 18/25 patients received adjuvant treatment, (stage II, *n* = 1; stage III, *n* = 3; stage IV, *n* = 14 at diagnosis) (Figure 1B, Table 2, Table S1). A response measurement was carried out in 15/18 patients. After adjuvant treatment, 15/18 patients had a progression or relapsed. The TTP for these 18 patients, if available, is shown in Figure S1B.

Patients with MiT‐RCC (*n* = 11) underwent five different adjuvant treatment approaches, of whom five were treated with sunitinib, resulting in stable disease (*n* = 4) and an unknown response (*n* = 1). In one instance the combination of sunitinib with radiotherapy led to progression of disease. Progression of disease was also seen after chemo‐immunotherapy (*n* = 1) and treatment with nivolumab plus ipilimumab (*n* = 1). However, nivolumab plus ipilimumab also led to stable disease (*n* = 1) and nivolumab monotherapy to a partial response (*n* = 1).

Next to the patients with MiT‐RCC, 5/18 patients with papillary RCC, 1/18 with RMC, and 1/18 with clear‐cell RCC received adjuvant treatment. Whereas sunitinib monotherapy often resulted in stable disease in MiT‐RCC, it led to progressive disease in four patients with papillary RCC (Figure 1B). In RMC, cisplatin, paclitaxel, and gemcitabine/bortezomib, followed by carboplatin and gemcitabine, were administered and this resulted in progressive disease. In the same patient but in the neo‐adjuvant setting, two similar chemotherapies, including bortezomib, led to a transient partial response (TTP: 3.7 months and 6.3 months) (Table S1). However, a patient with stage III clear‐cell RCC and a positive lymph node had a complete response after treatment with interleukin 2 (IL‐2). There is an ongoing disease‐free survival of 88 months (Figure 1B, Table S1).

### 3.7 Relapse and progression treatment and response

Thirty‐one different treatment approaches for relapse and/or progression were given in a group of 24 patients (Figure 1C, Table S1). Before relapse or progression, these patients either underwent only nephrectomy (*n* = 6), received neo‐adjuvant treatment (*n* = 10), and/or adjuvant treatment (*n* = 14). The TTP for these 24 patients is shown, if available, in Figure S1C. Within the MiT‐RCC group (*n* = 17), sunitinib was most frequently chosen, with three patients showing a partial response, four patients showing stable disease, and two patients having progressive disease. Alternative TKI monotherapies were axitinib (*n* = 5), cabozantinib (*n* = 4), pazopanib (*n* = 2), and sorafenib (*n* = 5). A partial response to cabozantinib, pazopanib, or sorafenib was observed in three patients. Aside from TKI treatment, everolimus and nivolumab monotherapy both resulted in progression of disease in four and five patients, respectively. However, the combination of nivolumab plus radiotherapy, and everolimus plus lenvatinib both induced a partial response.

Five patients with papillary RCC were treated with five different agents at relapse and/or progression. In three patients, treatment with nivolumab led to progressive disease. Treatment with axitinib and cabozantinib led to similar responses in two patients. The only drugs that induced a partial response was the combination of durvalumab plus tremelimumab.

Altogether, there were three patients who had a complete response (Figure 1C, Table S1). A stage IV MiT‐RCC patient showed a response to radiotherapy and continued nivolumab treatment for 17.5 months before progressing, after which a total nephrectomy was performed. Continuation of treatment with nivolumab after surgery led to the patient living without evidence of disease. A stage III MiT‐RCC patient, treated upfront with a complete nephrectomy and lymph node dissection, showed nodal relapse. After lymphadenectomy, the patient progressed again after 16.5 months. A complete response was acquired after 6 months of IL‐2 treatment before the next relapse, requiring multiple additional therapies (Table S1). Finally, a patient with stage I clear‐cell RCC with a relapse 22 months after nephrectomy showed no evidence of disease after lymphadenectomy and sunitinib for 56 months.

### 3.8 Frequently used approaches

**FIGURE 2:**
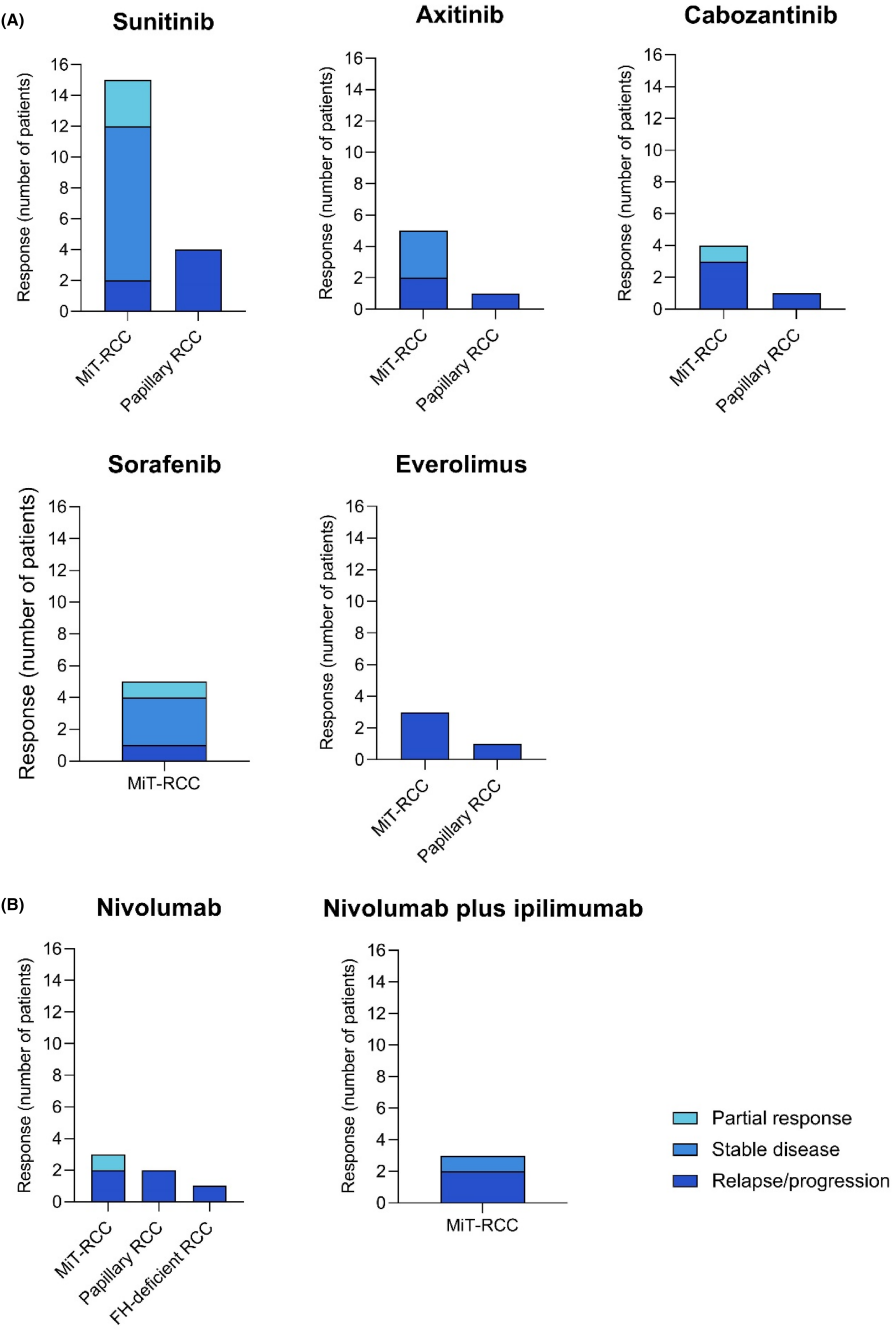
N describes the number of times a treatment approach was given. One treatment is therefore defined as the start of treatment with a certain drug or combination of drugs until a response measurement, leading to a new treatment approach based on the response measurement (i.e., treatment with a different drug). The best response was reported. In this figure, no distinction has been made between the neoadjuvant‐, adjuvant‐ and relapse/progression treatment. UC, unclassified. Clear, clear‐cell RCC. Pap, papillary RCC.

Although the treatment regimens were very heterogeneous and only given once, some targeted agents were recurrently administered. This included sunitinib, axitinib, cabozantinib, sorafenib, everolimus, nivolumab, and the combination of nivolumab plus ipilimumab (Figure 2). Overall, sunitinib was administered 19 times, with varying response. Stable disease was most seen in patients with MiT‐RCC (Figure 2A). Axitinib was given to six patients, resulting in stable disease in half of them. Cabozantinib and sorafenib induced similar responses in five patients each. By contrast, everolimus did not induce a response and led to progressive disease three times (Figure 2A). Similarly, nivolumab led to progressive disease in five patients and only induced a partial response in one patient (Figure 2B). The immuno‐combination therapy, consisting of nivolumab plus ipilimumab did not induce a response; however, stable disease was reported in one patient (Figure 2B).

### 3.9 Outcome

Overall, 15/31 patients died of disease (Table 1). Among these patients were 12 with stage IV and three with stage III disease at diagnosis. Twelve out of 31 patients are still alive with evidence of disease, and four patients are without evidence of disease (Table 1).

### 3.10 Toxicity

Adverse events, including their toxicity grade, were reported 109 times, allowing multiple registered adverse events per treatment approach (Table S2). There were no toxicity‐related deaths. High‐grade adverse events (grade III‐IV) were reported 38 times.

## 4 DISCUSSION

The administration of targeted drugs such as TKIs or PD‐(L)1 immunotherapy in treatment of pediatric RCC is not well‐documented. So far, only small series and case reports have been described in literature.^3^, ^22^ This retrospective descriptive study is the first study, initiated by the SIOP‐RTSG, that describes the response of advanced‐stage and relapsed pediatric patients with RCC treated with targeted agents. Some patients were previously described but for this study, detailed data were acquired.^9^, ^10^, ^29^ Thirty‐one patients with RCC were retrospectively identified through internationally collected CRFs.

In this selective cohort, it has been confirmed that pediatric RCC is mainly diagnosed during the second decade of life, in contrast to other pediatric renal tumors such as the frequently occurring Wilms tumor.^2^, ^17^, ^30^ The median age at diagnosis (11.4 years) of our subset of patients with RCC was consistent with previous findings from Van der Beek et al. and Indolfi et al., who reported a median age of 11.3 years (range 0.8–16.6 years) and 10.3 years, respectively, in relatively large cohorts.^4^, ^10^, ^31^

The majority of the patients presented with advanced‐stage disease. This was expected in this cohort as TKIs are rarely administered for localized disease, which should be approached with surgery alone, as shown by Geller et al.^9^, ^32^ Whereas previous SIOP‐RTSG protocols did not advocate a clear approach for treatment of advanced and metastatic RCC with TKIs and PD‐(L)1‐inhibitors, the current SIOP‐RTSG 2016 UMBRELLA protocol advises to administer sunitinib.^9^, ^22^ While in adults targeted therapies for RCC are rapidly being implemented, their application in the pediatric setting is much more cautious due to paucity of knowledge and rarity of pediatric RCC and the different distribution of subtypes in children.^1^, ^3^

TKI monotherapies that were given to pediatric patients in this study consisted of sunitinib, axitinib, cabozantinib, pazopanib, and sorafenib and as of late, there is a growing interest in these type of agents.^14^ These TKIs were given in metastatic cases as well as in a relapse and progression setting, and showed results varying from partial response to progressive disease. We acknowledge that current response data cannot be used to draw strong conclusions in this heterogeneous patient population, yet this is a first attempt to explore which newer agents might be worthy to investigate in the setting of pediatric RCC.

Within the different pediatric RCC subtypes, MiT‐RCC is the most prevalent, which was formally recognized by the WHO in 2004. There are multiple techniques available for subtype classification to check overexpression of TFE3 protein, indicative of TFE3 gene fusion, and suggesting MiT‐RCC histology.^26^ In most of patients with MiT‐RCC, diagnosis was confirmed through TFE‐assessment. MiT‐RCC rates similar to our findings were found in a review from Geller et al. with 8/11 pediatric patients having MiT‐RCC.^3^ Similarly, Van der Beek et al. reported MiT‐RCC in more than half of the pediatric patients in a SIOP‐RTSG study.^10^ By contrast, the AREN03B2 study with children and young adolescents reported MiT‐RCCs in 41.5% of 212 tumors, which can be attributed to the larger number of cases and the inclusion of additional patients with low‐stage disease who were not treated with targeted therapy.^2^ High‐stage tumors are more often classified as MiT‐RCC than low‐stage tumors according to a review by Geller et al.^3^ Besides the differences found between these two studies, the MiT‐RCC rates are still higher than in adults.^2^, ^3^, ^33^, ^34^

Since adult studies focusing on targeted therapies are often focused on clear‐cell RCC, the high number of pediatric MiT‐RCC cases, combined with an often advanced‐stage disease, indicates that this subtype might need a specific treatment approach.^4^, ^8^ The effect of targeted therapies that have been implemented in treatment for adults, mainly focusing on clear‐cell RCC, remains uncertain in the adjuvant setting in children.^8^ A phase III trial that compared sunitinib to interferon alpha in 750 adult patients found a better efficacy for sunitinib, which led to adoption of sunitinib as first‐line treatment of metastatic RCC.^22^, ^35^ Despite this evidence in adult RCC, the SIOP‐RTSG 2016 protocol has stressed the lack of evidence in the pediatric setting.^9^ In our cohort, responses in patients varied but disease was mostly stable, so until new evidence becomes available, sunitinib remains the standard of care following the 2016 UMBRELLA protocol.

This study contained only two pediatric patients with clear‐cell RCC, both reaching a complete response under IL‐2 as adjuvant therapy for stage III disease, and sunitinib with additional lymph node removal at relapse, respectively. In this cohort, most patients with papillary RCC had progressive disease after treatment with targeted agents. Altogether, no strong conclusions could be drawn regarding the treatment responses of these rarer pediatric RCC subtypes.

The SIOP‐RTSG UMBRELLA 2016 protocol recommends everolimus or immunotherapy for metastatic RCC when there is no response to sunitinib.^9^ Single‐agent immunotherapy or everolimus did not show convincing results in this cohort; however, recently, a partial response to nivolumab was reported in a 32 year‐old female with MiT‐RCC.^36^ In contrast to our results for single‐agent therapy, the combination of immunotherapy with another immunotherapeutic agent or a TKI led to a partial response in six instances (sunitinib plus sintilimab, axitinib plus pembrolizumab, axitinib plus avelumab, lenvatinib plus everolimus, and durvalumab plus tremelimumab). The first three combinations could potentially be an interesting focus for future research. Combinations of axitinib plus pembrolizumab, and axitinib plus avelumab led to a significantly longer PFS compared to sunitinib in an adult randomized phase III trial.^15^, ^35^ Overall, this may indicate that combination therapy could be of value in future studies, taking into consideration the potential toxicity of a combination of these agents. Comparison of toxicity profiles seemed to show no compound that had a particularly significant profile of adverse events compared to other agents.

Despite the novelty of this study, several limitations need to be addressed. Firstly, this was a retrospective study of a relatively small population, which is common in the setting of pediatric cancer, with highly varying treatment approaches. Treatment backgrounds of the patients were dissimilar and therefore difficult to compare. Furthermore, some targeted therapies were given at different points in treatment, and TTP data were limited. Given this highly variable data and the limitations in outcome data, this study could not reliably conclude on outcome data for separate drugs, and comparative analyses between patients with the same treatment should be interpreted with caution. Similarly, data collection was not complete for some patients due to ongoing treatment, which has impacted the analysis of response data and the RCC survival rate in this cohort. Finally, due to the retrospective nature of this study and the data‐capturing through internationally distributed CRFs, a conceivable registration bias and incomplete patient registration should be taken into consideration. On the contrary, we remark that this picture represents real life in terms of very heterogeneous decision‐making and choice of therapeutic approaches, which underlies the need for structured investigations in the future. In the adult literature, given the very large number of cases, many clinically controlled studies guide clear therapeutic algorithms. In the pediatric setting, it is likely that studies like ours might serve to identify a drug with treatment potential, in which to invest resources.

In conclusion, this study intended to describe the treatment approach and response of patients with pediatric RCC, registered in the SIOP (93‐01, 2001, UK‐IMPORT, and UMBRELLA) and AIEOP settings, who have been treated with targeted agents. This study stresses the importance of international registration of these patients, limiting heterogeneity in treatment of pediatric RCC with worse prognosis. Also, several drugs have been reported to have a good effect in adult RCC, and may be further analyzed in the pediatric setting.^15^, ^37^ A recently created pediatric tumor organoid biobank, including MiT‐RCC models, shows potential for drug sensitivity assays.^38^ Using MiT‐RCC organoids, we may be able to select potential therapies more effectively. Based on our findings, future studies need to focus on identifying potential beneficial treatment regimens and we would like to stress the importance of ongoing international collaboration.

## AUTHOR CONTRIBUTIONS

**Julia Sprokkerieft:** Conceptualization (equal); data curation (equal); formal analysis (equal); investigation (lead); methodology (equal); project administration (equal); resources (equal); supervision (equal); visualization (lead); writing – original draft (lead); writing – review and editing (lead). **Justine N. van der Beek:** Conceptualization (equal); data curation (equal); formal analysis (equal); investigation (lead); methodology (equal); project administration (equal); resources (equal); supervision (equal); visualization (lead); writing – original draft (lead); writing – review and editing (lead). **Filippo Spreafico:** Conceptualization (equal); investigation (lead); methodology (equal); resources (equal); visualization (equal); writing – original draft (equal); writing – review and editing (equal). **Barbara Selle:** Conceptualization (equal); investigation (lead); methodology (equal); resources (equal); visualization (equal); writing – original draft (equal); writing – review and editing (equal). **Estelle Thebaud:** Investigation (equal); resources (equal); writing – review and editing (equal). **Tanzina Chowdhury:** Conceptualization (equal); investigation (lead); methodology (equal); resources (equal); visualization (equal); writing – original draft (equal); writing – review and editing (equal). **Jesper Brok:** Investigation (equal); resources (equal); writing – review and editing (equal). **Gábor Ottóffy:** Investigation (equal); resources (equal); writing – review and editing (equal). **Xiaofei Sun:** Investigation (equal); resources (equal); writing – review and editing (equal). **Gema L. Ramírez Villar:** Investigation (equal); resources (equal); writing – review and editing (equal). **Garik Sagoyan:** Investigation (equal); resources (equal); writing – review and editing (equal). **Heidi Segers:** Investigation (equal); resources (equal); writing – review and editing (equal). **Dimitrios Doganis:** Investigation (equal); resources (equal); writing – review and editing (equal). **Annalisa Serra:** Investigation (equal); resources (equal); writing – review and editing (equal). **Lauriane Lemelle:** Investigation (equal); resources (equal); writing – review and editing (equal). **Norbert Graf:** Conceptualization (equal); investigation (lead); methodology (equal); resources (equal); visualization (equal); writing – original draft (equal); writing – review and editing (equal). **Arnauld Verschuur:** Conceptualization (equal); investigation (lead); methodology (equal); resources (equal); visualization (equal); writing – original draft (equal); writing – review and editing (equal). **Godelieve Tytgat:** Conceptualization (equal); formal analysis (equal); investigation (lead); methodology (equal); project administration (equal); resources (equal); supervision (equal); visualization (equal); writing – original draft (equal); writing – review and editing (equal). **Marry M. van den Heuvel‐Eibrink:** Conceptualization (equal); formal analysis (equal); investigation (lead); methodology (equal); project administration (equal); resources (equal); supervision (equal); visualization (equal); writing – original draft (equal); writing – review and editing (equal).

## CONFLICT OF INTEREST STATEMENT

The authors declare no conflict of interest.

## ETHICS STATEMENT

This study was approved by the SIOP‐RTSG steering committee in 2020. The requirement for ethical approval was waived due to the retrospective nature of this study and embedment in the SIOP 93–01, SIOP 2001 (SIOP WT 2001 trial, EudraCT number: 2007–004591–39), UMBRELLA 2016 (EudraCT number: 2016–004180‐39), UK‐IMPORT (London Bridge number: 12/LO/0101) and AIEOP studies. Informed consent from registration in these studies had been obtained from parents of included pediatric patients prior to treatment, according to national law and regulations.

## Supporting information


Appendix S1

## Data Availability

The data that support the findings of this study are available in the Supplementary Material and from the SIOP‐RTSG office following standard access procedures upon reasonable request.
