# Supplementary material for: Targeted therapies in children with renal cell carcinoma (RCC): An International Society of Pediatric Oncology—Renal Tumor Study Group (SIOP‐RTSG)‐related retrospective descriptive study
Source: Cancer Med. 2023 Dec 22;13(1):e6782. doi: 10.1002/cam4.6782 (PMC10807685; doi:10.1002/cam4.6782)
Supplement: Supplementary file 1 — Appendix S1 [file CAM4-13-e6782-s001.docx]

|  | | **Supplementary table 1.** Description of treatment, response and outcome with pre-operative, post-operative and relapse or progression treatment. | | | | | | | | | | | | |
| --- | --- | --- | --- | --- | --- | --- | --- | --- | --- | --- | --- | --- | --- | --- |
| Patient # | Stage at  diagnosis | | Histology at  diagnosis | Neo-adjuvant treatment | | | Surgery | Adjuvant treatment | | | Relapse/progression treatment | | | Outcome |
|  |  |  |  | Treatment | Time-to-progression | Response |  | Treatment | Time-to-progression | Response | Treatment | Time-to-progression | Response |  |
| 1 | II | | MiT-RCC | - | - | - | Total nephrectomy | Sunitinib | - | SD | LN resection  Surrenalectomy +  LN resection | -  - | -  - | ED |
| 2 | II | | MiT-RCC | - | - | - | Total nephrectomy | - | - | - | LN resection  Vinblastine + interferon  Sunitinib  Radiotherapy + neurosurgery  Sorafenib | -  10,5m DR  3m DR, 6m  -  18m DR | -  PR  PR  -  PR | ED |
| 3 | III | | MiT-RCC | - | - | - | Total nephrectomy | LN resection | - | SCR | LN resection  Splenectomy  IL-2  Sunitinib  Axitinib + radiotherapy  Everolimus | 16.5m  -  8.4m  18.6m  2m  - | SCR  -  CR  PD  PD  PD | DOD |
| 4 | III | | MiT-RCC | - | - | - | Total nephrectomy | - | - | - | Axitinib + pembrolizumab | - | PR/CR | ED |
| 5 | III | | MiT-RCC | - | - | - | Total nephrectomy | - | - | - | Sunitinib  Radiotherapy  Everolimus | 3m  -  1m | PR  -  PD | DOD |
| 6 | III | | MiT-RCC | - | - | - | Total nephrectomy | - | - | - | Nivolumab + ipilimumab  Cabozantinib | 9m  12m | PD  PD | ED |
| 7 | III | | MiT-RCC | - | - | - | Total nephrectomy | - | - | - | Sunitinib  Nivolumab + capecitabine  Chemotherapy ^b^ | 39d  56d  41d | PD  PD  PD | DOD |
| 8 | III | | MiT-RCC | - | - | - | Total nephrectomy | Nivolumab + ipilimumab  Nivolumab | -  - | SD  PR | - | - |  | NED |
| 9 | III | | MiT-RCC | Nivolumab + ipilimumab | - | *NS* | Pre-op setting | - | - | - | - | - | - | ED |
| 10 | IV | | MiT-RCC | - | - | - | Total nephrectomy | Sunitinib + radiotherapy | 3.5m | PD | Sunitinib + radiotherapy  Sorafenib  Axitinib | 3m  9m  - | PD  SD  PD | DOD |
| 11 | IV | | MiT-RCC | Chemo-therapy ^a^ | 0.7m | PD | Total nephrectomy | Chemo-immuno-therapy ^f^ | 2m | PD | Chemotherapy ^h^  Chemotherapy ^i^  Chemotherapy ^j^  Sunitinib  Allo-SCT + vaccination  Sunitinib  Interferon $\alpha$2a  Sorafenib  Axitinib  Resection metastases  Sorafenib + everolimus + nivolumab | 1.4m  -  -  -  -  -  4m  -  68m  19m  2m | PR  SD  SD  SD  -  SD  PD  SD  PD  -  PD | DOD |
| 12 | IV | | MiT-RCC | - | - | - | Total nephrectomy | Sunitinib | 10m | SD | Nivolumab >  nivolumab + ipilimumab  Axitinib  Cabozantinib  Everolimus  Axitinib + everolimus  Pazopanib  Vinorelbine | 5m  10m  3m  3m  2.5 – 5.5m  0.75m  - | SD  SD  PD  PD  SD  PD  PD | DOD |
| 13 | IV | | MiT-RCC | - | - | - | Total nephrectomy | Nivolumab + ipilimumab | 3.9m | PD | Cabozantinib  Lenvatinib + everolimus | -  - | PR  PR | ED |
| 14 | IV | | MiT-RCC | Chemo-therapy ^b^ | 1.5m | PD | Total nephrectomy | - | - | - | Sunitinib | 4.5m | SD | DOD |
| 15 | IV | | MiT-RCC | Sunitinib | 6.5m | SD | Total nephrectomy | Sunitinib | 6.5m | SD | Cabozantinib  Nivolumab | 3m  3.3m | PD  PD | DOD |
| 16 | IV | | MiT-RCC | - | - | - | Total nephrectomy | Sunitinib | NS | NS | Post-op setting | - | - | ED |
| 17 | IV | | MiT-RCC | Sunitinib | - | - | Total nephrectomy | Sunitinib | - | SD | Radiotherapy  Sunitinib  Nivolumab | -  2.5m  2.5m | -  SD  PD | ED |
| 18 | IV | | MiT-RCC | Sunitinib | 11m | SD | No surgery | - | - | - | Radiotherapy > nivolumab  Complete nephrectomy > nivolumab | 17.5m DR  - | SD/PR  SCR | NED |
| 19 | IV | | MiT-RCC | Axitinib + avelumab | - | PR | Bilateral total nephrectomy | Radiotherapy | - | *NS* | Resection lesion  Radiotherapy + axitinib + avelumab | -  - | *NS*  *NS* | ED |
| 20 | IV | | MiT-RCC | Sunitinib + sintilimab | 3.6m | PR | No surgery | - | - | - | - | - | - | DOD |
| 21 | IV | | MiT-RCC | Chemo-therapy ^c^ | - | SD | No surgery | - | - | - | Sunitinib  Sorafenib  Sorafenib (lower dose)  Axitinib  Embolization  Pazopanib  Radiotherapy  Axitinib  Radiotherapy  Sorafenib  Nivolumab | 4m  42m  6m  -  -  10m  10m  -  7m  -  2m | PR  SD  SD  SD  -  PR  -  SD  -  PD  - | DOD |
| 22 | IV | | Papillary | Sunitinib | - | *NS* | Total nephrectomy | Sunitinib | 1.2m | PD | Axitinib  Nivolumab | 1m  0.3m | PD  PD | DOD |
| 23 | IV | | Papillary | - | - | - | Total nephrectomy | TKI (*NS)* | - | PR | TKI (*NS)* | - | PR | DOD |
| 24 | IV | | Papillary | - | - | - | Total nephrectomy | Sunitinib | 10m | PD | Durvalumab + tremelimumab | 13m DR | PR | ED |
| 25 | IV | | Papillary | - | - | - | Total nephrectomy | Sunitinib | 8.5m | PD | Nivolumab  Cabozantinib  Everolimus  Surgery > radiotherapy > axitinib | 4m  8.3m  1.5m  1m | PD  PD  PD  PD | DOD |
| 26 | IV | | Papillary | - | - | - | *NS* | Sunitinib | 2.8m | PD | Nivolumab | - | - | ED |
| 27 | IV | | RMC | Chemo-therapy ^d^  - | 3.7m  6.3m | PR  PR | Total nephrectomy | Chemo-therapy ^g^ | 1.4m | PD | Bevacizumab + erlotinib  Chemotherapy ^g^ | 4.7m  6m | -  PR | ED |
| 28 | IV | | RMC | Chemo-therapy ^e^ | 7.5m | PR | No surgery |  |  |  | Nivolumab  Radiotherapy | 3m  - | PD  - | DOD |
| 29 | I | | Clear cell | - | - | - | Total nephrectomy | - | - | - | LN resection + sunitinib | 87.6m DR | CR | NED |
| 30 | III | | Clear cell | - | - | - | Total nephrectomy | IL-2 | 88.4m DR | CR | - | - | - | NED |
| 31 | IV | | FH deficient | Axitinib + avelumab | - | PR | Pre-op setting | - | - | - | - | - | - | ED |
| CR = complete response; PR = partial response; MR = mixed response; SD = stable disease; PD = progressive disease; NED = no evidence of disease; ED = evidence of disease; DOD = dead of disease; MiT-RCC = translocation type renal cell carcinoma; RMC = renal medullary carcinoma; FH deficient = fumarate hydratase deficient RCC; LN resection = lymph node resection; PBSCT = peripheral blood stem cell transplantation; *NS* = not specified. ^a^ Vincristine, actinomycin-D, doxorubicin. ^b^ Irinotecan and olaparib. ^c^ Gemcitabine and oxaliplatin. ^d^ Cisplatin, paclitaxel and doxorubicin; cisplatin, gemcitabine, ifosfamide; cisplatin, paclitaxel, gemcitabine or bortezomib, carboplatin and gemcitabine. ^e^ Cisplatin, paclitaxel and gemcitabine. ^f^ Capecitabine, isotretinoin plus interleukin-2, interferon-α2a and DGCiN98. ^g^ Cisplatin, paclitaxel and gemcitabine/bortezomib, carboplatin and gemcitabine. ^h^ Ifosfamide, cisplatin and doxorubicin. ^i^ Topotecan + carboplatin. ^j^ Cisplatin, 5-Fluorouracil. | | | | | | | | | | | | | | |


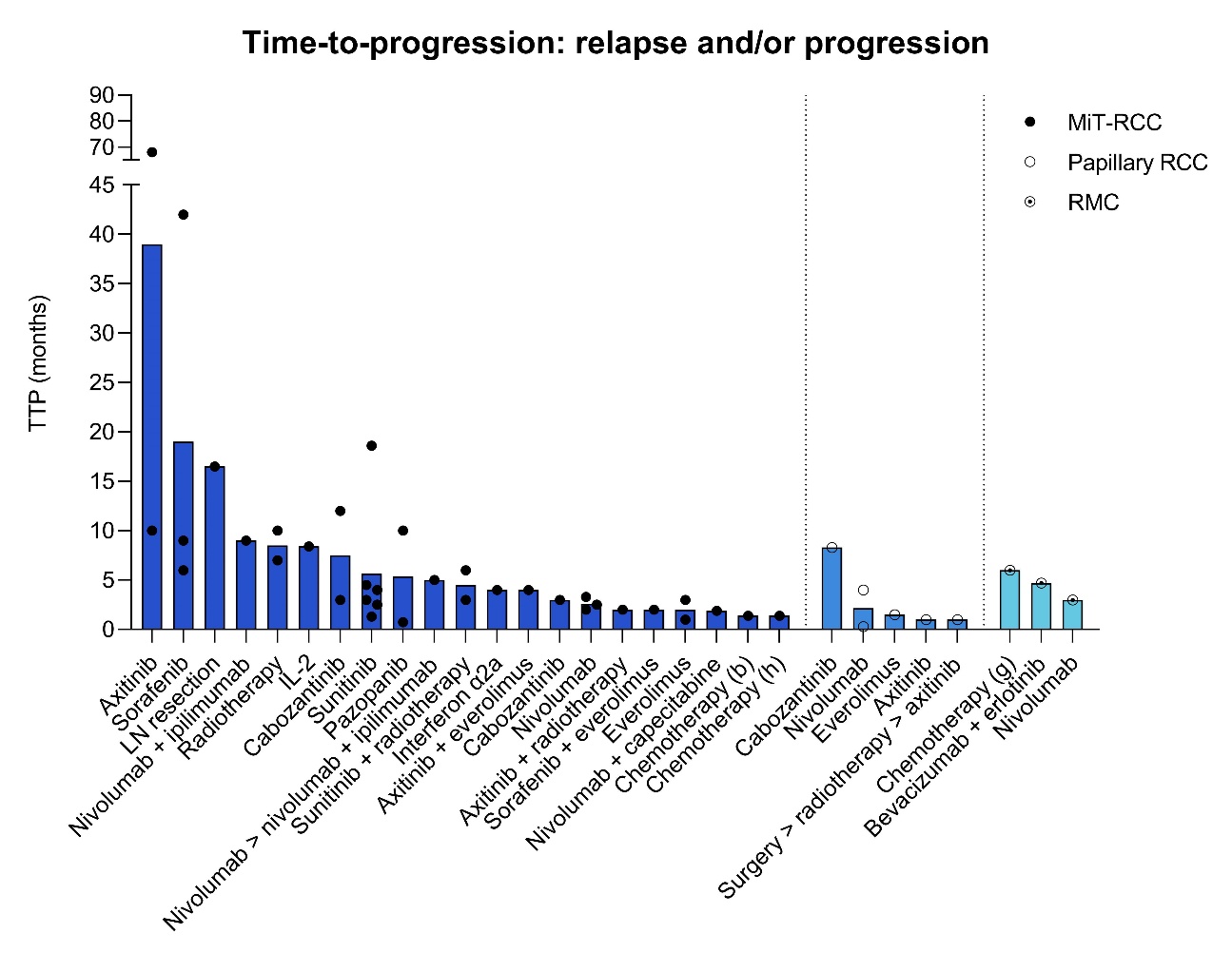

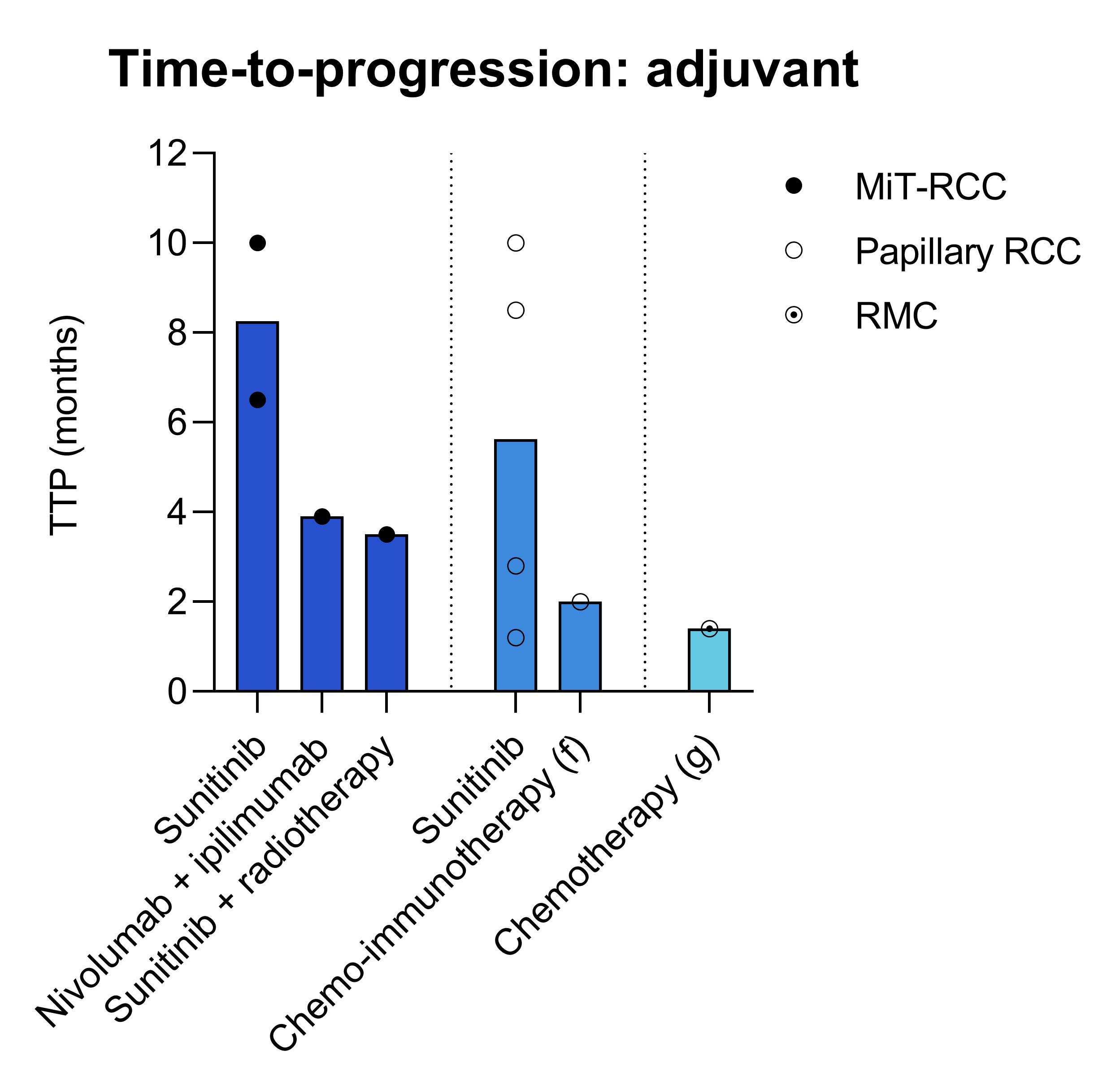

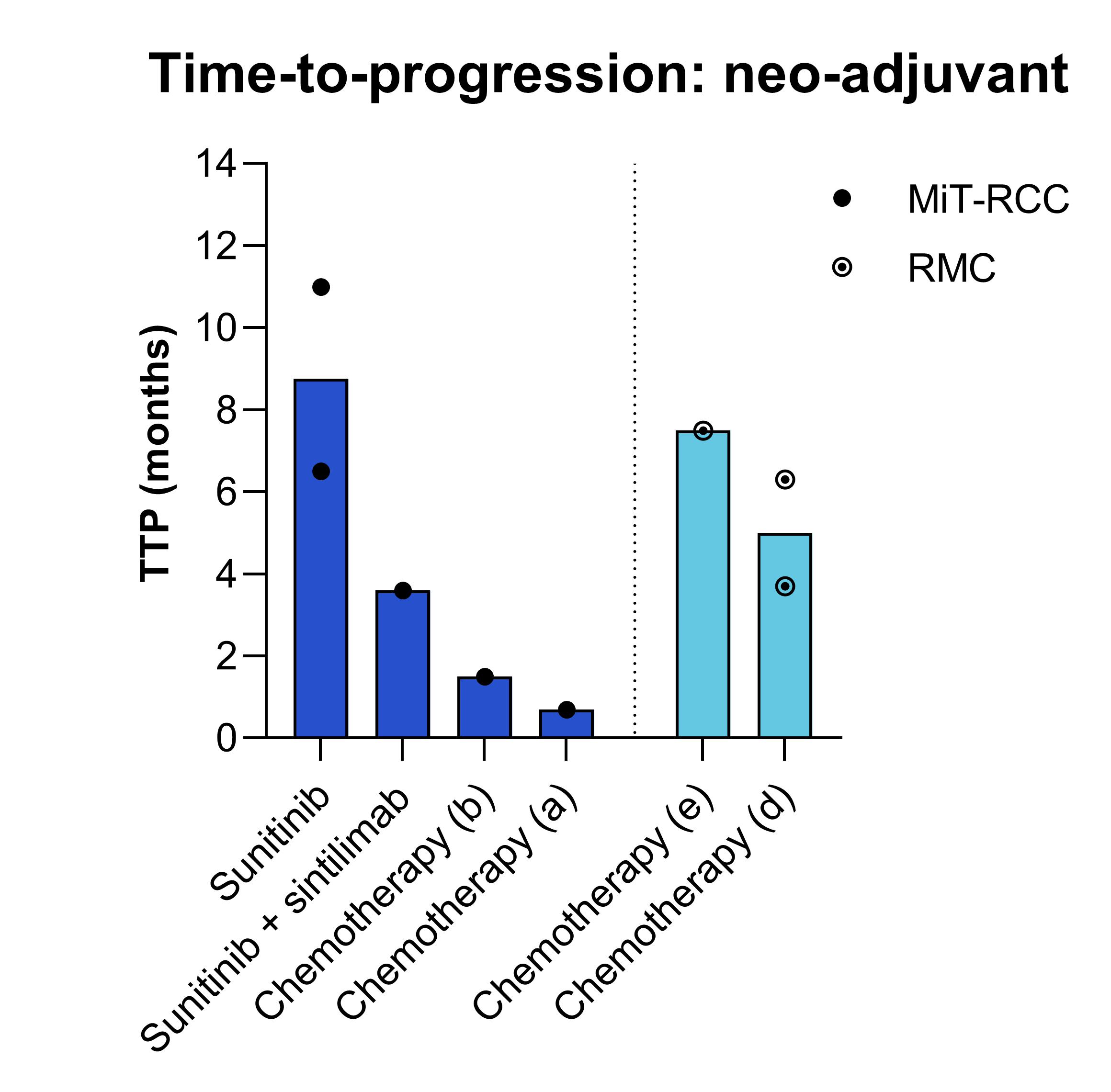


**A**

**B**

**C**

**Supplementary figure 1.** The mean time-to-progression in the (A) neoadjuvant setting, (B) adjuvant setting and (C) relapse and/or progression setting.

TTP = time-to-progression. ^a^ Vincristine, actinomycin-D, doxorubicin. ^b^ Irinotecan and olaparib. ^d^ Cisplatin, paclitaxel and doxorubicin; cisplatin, gemcitabine, ifosfamide; cisplatin, paclitaxel, gemcitabine or bortezomib, carboplatin and gemcitabine. ^e^ Cisplatin, paclitaxel and gemcitabine. ^f^ Capecitabine, isotretinoin plus interleukin-2, interferon-α2a and DGCiN98. ^g^ Cisplatin, paclitaxel and gemcitabine/bortezomib, carboplatin and gemcitabine. ^h^ Ifosfamide, cisplatin and doxorubicin.

| **Supplementary table 2.** The occurrence of adverse events. N describes the number of times a treatment approach has induced an adverse event. | | | | | | | | | |  |
| --- | --- | --- | --- | --- | --- | --- | --- | --- | --- | --- |
| Drug | Grade not specified | Occ. | Grade I | Occ. | Grade II | Occ. | Grade III | Occ. | Grade IV | Occ. |
| Sunitinib (N=11) | Gastro-intestinal  Asthenia  Diarrhea  Hypertension  Molestias gastricas  Hand-foot syndrome  Grade I-II oral mucositis  Grade I-II fatigue  Grade III and IV anemia  Hair discoloration  Depigmentation  Hypothyroidism  Heart insufficiency  Arterial hypertension  Hand-foot syndrome | 1  1  1  1  1  1  1  1  1  1  1  1  1  1  1 | Gastro-intestinal  Nausea  Cutaneous effect | 1  1  1 | Hematological  Hypertension  Hypothyroidism  Hand-foot syndrome  Neutropenia (25mg/day) | 1  1  2  1  1 | Increased creatine and subsequent liver failure  Hematological | 1  1 | Anemia | 1 |
| Sunitinib + sintilimab (N = 1) | Gastro-intestinal | 1 | - | - | Infectious toxicity | 1 | - | - | - |  |
| Axitinib (N=2) | Headache  Abdominal pain – constipation  TSH elevation  CPK increase | 1  1  1  1 | Neutropenia | 1 | - | - | - | - | - |  |
| Axitinib + avelumab (N = 2) | Febrile neutropenia without ICU admission | 2 | - | - | - | - | - | - | - |  |
| Axitinib + pembrolizumab (N=1) | Febrile neutropenia with ICU admission  Dermatosis  Hypothyroidism  Hypertension  Alopecia  Interstitial nephritis | 1  1  1  1  1  1 | - | - | - | - | - | - | - |  |
| Cabozantinib (N=6) | Grade I-II myalgia  Neuropathic pain and neck pain  Asthenia  Abnormal pain in limbs and pelvis  Discoloration of hair  Peeling skin  Hypothyroidism | 1  1  1  1  2  1  1 | Gastro-intestinal | 1 | Gastro-intestinal  Hematological  Hand-foot syndrome  Hypothyroidism  Anemia  Nausea | 1  1  1  1  2  1 | Gastro-intestinal  Palmar-Plantar erythrodysesthesia | 1  1 |  |  |
| Bortezomib + chemotherapy  (N = 1) | Febrile neutropenia  Hematological  Infectious | 1  1  1 | - | - | - | - | - | - | - |  |
| Irinotecan + olaparib (N=1) | - | - | - | - | Infectious | 1 | Hematological  Gastro-intestinal | 1  1 | - |  |
| Pazopanib (N=2) | - | - | - | - | Anemia  Hyperproteinemia | 1  1 | - | - | - |  |
| Sorafenib (N=4) | Growth retardation  Grade II/II hand-foot syndrome  Grade II/III diarrhea  Anorexia  Hypothyroidism  Impaired growth  Erythematous and itchy patches w/o blood pressure disorder  Headache  Vesicular rash  Diarrhea  Nausea | 1  1  1  1  1  1  1  1  1  1  1 | Gastro-intestinal  Cutaneous | 2  1 | Gastro-intestinal  Proteinuria  Hypothyroidism | 1  1  1 | Hypertension | 1 |  |  |
| Everolimus (N=1) | Mouth ulcer | 1 |  |  |  |  |  |  |  |  |
| Nivolumab (N=4) | Diarrhea  Hypothyroidism  Erythematous and itchy patches w/o blood pressure disorder | 1  1  1 | - | - | - | - | DD pneumonitis with hypoxia  Hematological | 1  1 | - |  |
| Nivolumab + Ipilimumab (N=1) | Diarrhea  Vesicular rash  Nausea  Headache  Diarrhea | 2  1  1  1  1 | - | - | - | - | - | - | - |  |
| IL-2 (N = 1) | Thyroid gland | 1 | Gastro-intestinal  Nausea | 1  1 | - | - | - | - | - |  |

Occ = occurrence.
